# A Genetic Investigation of the Well-Being Spectrum

**DOI:** 10.1007/s10519-019-09951-0

**Published:** 2019-02-27

**Authors:** B. M. L. Baselmans, M. P. van de Weijer, A. Abdellaoui, J. M. Vink, J. J. Hottenga, G. Willemsen, M. G. Nivard, E. J. C. de Geus, D. I. Boomsma, M. Bartels

**Affiliations:** 1 Department of Biological Psychology, Vrije Universiteit Amsterdam, Van der Boechorststraat 1, 1081 BT Amsterdam, The Netherlands; 2 Amsterdam Public Health Research Institute, Amsterdam, The Netherlands; 3 Behavioural Science Institute, Radboud University, Nijmegen, The Netherlands; 4 Neuroscience Amsterdam, Amsterdam, The Netherlands; 5 Department of Psychiatry, Amsterdam University Medical Centre, Location Academic Medical Center, Amsterdam, The Netherlands

**Keywords:** Well-being spectrum, Personality, Genetic correlation, Loneliness, Self-rated health, Flourishing

## Abstract

The interrelations among well-being, neuroticism, and depression can be captured in a so-called well-being spectrum (3-phenotype well-being spectrum, 3-WBS). Several other human traits are likely linked to the 3-WBS. In the present study, we investigate how the 3-WBS can be expanded. First, we constructed polygenic risk scores for the 3-WBS and used this score to predict a series of traits that have been associated with well-being in the literature. We included information on loneliness, big five personality traits, self-rated health, and flourishing. The 3-WBS polygenic score predicted all the original 3-WBS traits and additionally loneliness, self-rated health, and extraversion (R^2^ between 0.62% and 1.58%). Next, using LD score regression, we calculated genetic correlations between the 3-WBS and the traits of interest. From all candidate traits, loneliness and self-rated health were found to have the strongest genetic correlations (*r*_g_ = − 0.79, and *r*_g_= 0.64, respectively) with the 3-WBS. Lastly, we use Genomic SEM to investigate the factor structure of the proposed spectrum. The best model fit was obtained for a two-factor model including the 5-WBS traits, with two highly correlated factors representing the negative- and positive end of the spectrum. Based on these analyses we propose to include loneliness and self-rated health in the WBS and use a 5-phenotype well-being spectrum in future studies to gain more insight into the determinants of human well-being.

**Electronic supplementary material:**

The online version of this article (10.1007/s10519-019-09951-0) contains supplementary material, which is available to authorized users.

## Introduction

Many psychiatric disorders share a common genetic liability (Bramon and Sham 2001; Koenen et al. 2008; Peerbooms et al. 2011). This common genetic liability offers an explanation as to why many disorders are comorbid or represent highly similar behaviours. While there have been detailed investigations of the genetic similarity and comorbidity of psychiatric disorders, there is much less information about the genetic similarity of mental health-related traits such as happiness, satisfaction with life, personality, loneliness, self-rated health, and flourishing. Studies on traits that could be considered to be part of a well-being spectrum are important given the large collection of studies pointing to the emotional, cognitive, and interpersonal benefits of high levels of well-being above and beyond the absence of disorders (Keyes 2002; Diener and Chan 2011; Chmiel et al. 2012). It is also for that reason that the world health organization (WHO) defines health as “a state of complete physical, mental and social well-being and not merely the absence of disease or infirmity”. Therefore, the aim of this study was to investigate the genetic similarity between several traits associated with well-being, collectively referred to as the “well-being spectrum”.

Well-being is a broad and complex construct used to describe optimal psychological functioning (Ryan and Deci 2001). While several definitions exist, well-being research in the social sciences is often divided over two definitions: subjective/hedonic well-being (SWB) and psychological/eudaimonic well-being (PWB) (Ryan and Deci 2001). While there is some inconsistency in the use of these two terms, SWB is mostly viewed as a global evaluation of life satisfaction, while PWB is concerned more with human development and the realization of goals (Keyes et al. 2002). Recently, it has been proposed to use a data-driven spectrum approach to study well-being (Baselmans et al. 2019). This well-being spectrum (the 3-phenotype well-being spectrum; 3-WBS) captures the phenotypic and genetic overlap between subjective well-being, neuroticism, and depressive symptoms, as has been found in a large genome-wide association study (Okbay et al. 2016). There are nonetheless other traits that could be considered candidates for inclusion in a broader well-being spectrum. From a phenotypic perspective, it is important to identify such traits in order to get more insight into the aspects influencing human well-being. From a genetic perspective, it is important to identify these traits since their inclusion into the spectrum will help identify more genetic variants that influence human well-being.

One of the associations that has been studied thoroughly is the relationship between well-being and personality. Especially extraversion and conscientiousness have been established as strong positive phenotypic correlates of well-being (Soto 2015), while neuroticism has been identified as an important negative correlate of well-being (DeNeve and Cooper 1998). Furthermore, existing literature has established that loneliness, characterized by a sense of emptiness, worthlessness, and a lack of control (Cacioppo et al. 2010), is negatively associated with well-being (Ben-Zur 2012). Moreover, self-rated health, a subjective evaluation of one’s current health status, has also been pointed out as an important predictor of well-being, due to its high proportion of shared variance with well-being (Larson 1978; Okun and George 1984). Lastly, while the 3-WBS has included subjective/ hedonic well-being measures (such as satisfaction with life and subjective happiness), it did not yet include psychological or eudaimonic well-being measures, a well-being domain that involves the fulfilment of human potential (Ryan and Deci 2001). An example of such a measure is flourishing: a person’s self-perceived success in several life areas. Previous research on the relationship between psychological/eudaimonic- and subjective/hedonic well-being have revealed that these two lines of research reflect highly correlated, yet distinguishable constructs (Kashdan et al. 2008; Baselmans and Bartels 2018). Therefore, including both types of well-being could theoretically yield a more integrated conceptualization of the well-being spectrum.

Contrary to the phenotypic associations, few studies have investigated the genetic associations between well-being and well-being related traits. A genetic investigation of loneliness (Abdellaoui et al. 2018) revealed a strong negative association between a polygenic score for loneliness and subjective well-being, and a positive association with neuroticism and depression, indicating genetic links between the 3-WBS and loneliness. With regard to self-rated health, twin studies have demonstrated that both genes and the environment contribute to the association with well-being (Roysamb et al. 2003), but to our knowledge no molecular genetic study has been conducted yet. A twin study on the relationship between SWB and PWB indicates a single, genetic factor that accounts for the high heritability in both these constructs (Keyes et al. 2010). Likewise, a genome-wide association study on hedonic and eudaimonic well-being showed that there is a large overlap in the sets of genes influencing these two traits (Baselmans and Bartels 2018). Lastly, extraversion, and conscientiousness show not only strong phenotypic, but also genetic associations with well-being (Weiss et al. 2008, 2016).

In this study, we aim to further investigate the well-being spectrum from a genetic perspective. We perform three different types of genetic analyses to assess the genetic relatedness between the 3-WBS (subjective well-being, depressive symptoms, and neuroticism), and the likely candidates (loneliness, openness to experience, conscientiousness, extraversion, agreeableness, self-rated health, and flourishing). First, we use summary statistics from a large multivariate genome-wide association meta-analysis (GWAMA) of the 3-WBS (Baselmans et al. 2019) to calculate polygenic risk scores to predict the original 3-WBS and candidate traits as stated above. Since the amount of variance explained by polygenic scores can be small even though two traits are highly genetically correlated, we also calculate the standardized proportion of the variance shared by the traits that can be attributed to genetic factors, known as the genetic correlation, using LD score regression. Lastly, to get more insight into the factor structure of the genetically related traits, we applied Genomic SEM, a novel method for factor analysis using summary statistics only (Grotzinger et al. 2018).

## Materials and methods

### Participants

Participants are voluntary participants in the studies of the Adult Netherlands Twin Register (ANTR) (Boomsma et al. 2006; Willemsen et al. 2013). Participants were included if they had filled out questionnaires on one or more of the relevant traits and provided a blood or buccal cell sample for DNA isolation and genotyping. Based on the availability of the data, sample size per analysis varied. An overview of the sample characteristics can be found in Table 1 and details are provided below. The distribution of all phenotypic variables can be found in the Online Resource 1.

**Table 1:** Sample characteristics

Trait	Age *M* (SD)	*N* participants (% males)	Score *M* (SD)
Satisfaction with life	40.94 (15.83)	5344 (37.18%)	26.96 (4.70)
Happiness	39.36 (15.59)	5350 (37.14%)	22.48 (4.17)
Neuroticism	42.09 (15.94)	8622 (36.29%)	22.21 (8.10)
Depressive symptoms	38.68 (16.00)	8667 (36.45%)	3.58 (3.19)
Loneliness	43.38 (16.37)	8817 (36.43%)	3.82 (1.03)
Openness to experience	42.09 (15.94)	8622 (36.29%)	29.65 (6.58)
Conscientiousness	42.09 (15.94)	8622 (36.29%)	37.96 (6.28)
Extraversion	42.09 (15.94)	8622 (36.29%)	34.27 (6.73)
Agreeableness	42.09 (15.94)	8622 (36.29%)	37.42 (5.98)
Self-rated health	38.44 (15.68)	8667 (39.26%)	4.07 (0.59)
Flourishing	40.16 (14.96)	2200 (35.95%)	46.84 (6.47)

### The 3-WBS

For the 3-WBS, we used summary statistics from the N-weighted multivariate GWAMA by Baselmans et al. (2019). These summary statistics were computed by leveraging published univariate meta-analyses on life satisfaction, positive affect, neuroticism and depressive symptoms, adding up to a total of *N* = 2,370,390 observations. Levering so many data inevitably leads to sample overlap, which bias the resulting test statistics if not corrected properly. To this end, potential error correlation due to sample overlap between different meta-analyses was accounted for by estimating the dependence between effect sizes using LD score regression (Bulik-Sullivan et al. 2015).

### Subjective well-being—satisfaction with life

Satisfaction with life was assessed using the satisfaction with life scale (Diener et al. 1985) (inter-interviewer correlation = .73). The satisfaction with life scale contains 5 items measuring global cognitive judgments of satisfaction with one’s life on a scale from 1 (strongly disagree) to 7 (strongly agree). Items were summed to calculate an individual’s final score ranging from 0 to 35. A mean was calculated when satisfaction with life was assessed on more than one occasion. In total, data on satisfaction with life were available for 5344 individuals.

### Subjective well-being—happiness

Happiness was assessed using an adaptation of the subjective happiness scale (Cronbach’s α = 0.79–0.94) (Lyubomirsky and Lepper 1999). The adapted subjective happiness scale contains four items measuring global subjective happiness on a scale from 1 (strongly disagree) to 7 (strongly agree). Items were summed to calculate an individual’s final score, ranging from 0 to 28. A mean was calculated when subjective happiness was assessed on more than one occasion. In total, data on subjective happiness were available for 5350 individuals.

### Depressive symptoms

Depressive symptoms were assessed using the DSM-oriented depressive problem scale of the Adult Self Report (Cronbach’s α = 0.82) (Achenbach and Rescorla 2003). This scale contains 14 items measuring depression symptoms on a scale from 0 to 2 (0 = not true, 1 = somewhat true, 2 = very true or often true). The items were summed to create a sum score ranging from 0 to 28, a higher score representing higher levels of depressive symptoms. A mean was calculated when the depression problems were assessed on more than one occasion. In total, data on depressive symptoms were available for 8667 participants.

### Loneliness

Loneliness was assessed using the short scale for assessing loneliness in large epidemiological studies (Cronbach’s α = 0.72) (Hughes et al. 2004; Distel et al. 2010). This scale contains 3 items from the R-UCLA loneliness scale and asks participants to score how often they identify with the items on a scale from 1 to 3 (1 = hardly ever, 2 = some of the time, 3 = often). The items were summed to obtain a sumscore with possible scores between 3 and 9, a higher score representing higher levels of loneliness. In total, data on loneliness were available for 8817 participants. A mean was calculated when loneliness was assessed on more than one occasion. We log-transformed the loneliness scores since they were highly positively skewed.

### Personality

The Big Five personality traits were measured using the NEO-FFI (Costa and McCrae 1992; Franić et al. 2014). This scale measures the Big Five personality traits, openness to experience (α = 0.57–0.76), conscientiousness (α = 0.69–0.81), extraversion (α = 0.73–0.81), agreeableness (α = 0.66–0.70), and neuroticism (α = 0.80–0.88), with 60 items in total. Participants were asked to respond on a 5-point scale, ranging from 1 (strongly disagree) to 5 (strongly agree). The 12 items per trait were summed to obtain one sumscore for each personality trait with possible scores between 12 and 60, a higher score representing higher levels of that particular personality trait. When personality data were available for more than one occasion, we calculated an individual’s mean personality score per scale. In total, data on each personality scale were available for 8622 individuals.

### Self-rated health

Self-rated health was assessed using a single item: “How, in general, is your health?” (Eriksson et al. 2001). The item was rated on a 5-point scale, on which participants could respond with: “Bad”, “Poor”, “Fair”, “Good” or “Excellent”. A mean was calculated when Self-rated health was assessed on more than one occasion. In total, 8667 participants had data available for self-rated health.

### Flourishing

Flourishing was assessed using the Flourishing Scale (Cronbach’s α = 0.87) (Diener et al. 2010). This scale contains 8 items measuring a person’s self-perceived success in multiple life domains on a scale from 1 to 7, ranging from strong disagreement to strong agreement. The items were summed to create a sumscore ranging from 8 to 56, a higher score representing higher levels of positive flourishing. In total, data on flourishing were available for 2200 participants.

### Genotyping, quality control, imputation, and PCA

Genotyping was done on several genome-wide single nucleotide polymorphism (SNP) micro-arrays (Willemsen et al. 2013). Genotyped data were cross-platform imputed using the Genome of the Netherlands (GoNL) (Boomsma et al. 2014; The Genome of the Netherlands Consortium 2014) as a reference set to infer the SNPs missing per platform in the combined data (Fedko et al. 2015). Alleles with reference set allele frequency differences of > 10%, SNPs with minor allele frequency (MAF) < 0.005, deviation from Hardy–Weinberg Equilibrium (HWE) with p < 10^−12^, and a genotyping call rate < 0.95 were excluded for pre-imputation quality control. Samples that had a genotyping call rate < 0.90, inbreeding coefficient from PLINK (F) < − 0.075 or > 0.075 (Purcell et al. 2007), Affymetrix Contrast Quality Control metric < 0.40, Mendelian error rate > 5 standard deviations from the mean, or gender or Identity-by-State status that did not agree with known relationship status and genotypic assessment were excluded. MaCH-Admix software (Liu et al. 2013) was used for phasing and imputation. SNPs that were significantly associated with genotyping platform (*p* < 10^−5^), that had an allele frequency difference of > 10% with GoNL reference set, HWE p < 10^−5^, Mendelian error rate > 5 SD from the mean over all markers, or an imputation quality *R*^2^ < 0.90 after imputation were excluded. In order to exclude individuals with a non-Dutch ancestry and to control for Dutch population stratification, we performed Principal Components Analysis (PCA) following procedures described by Abdellaoui et al. (2013). The remaining SNPs (*N* = 1,224,793) were used to construct polygenic scores.

### Phenotypic correlations

Phenotypic correlations were calculated between all the traits using the gee package to correct for familial relatedness using R statistical software (R Core Team 2013). The results were visualized using the corrplot package. The significance threshold for the phenotypic correlations was set at a Bonferronni corrected value of α = 0.005/55 = 0.00009, where 55 represents the number of correlations that were calculated in total. To prevent the occurrence of false positives, we applied a baseline alpha of 0.005 for all analyses, in line with the reasoning of Benjamin and colleagues (Benjamin et al. 2018).

### Power analysis

We used an online power-calculator based on code provided by Dudbridge (Dudbridge 2013; Palla and Dudbridge 2015) to investigate whether the 3-WBS summary statistics (Baselmans et al. 2019) had sufficient power to predict the phenotypes that are considered to become part of the well-being spectrum. The power was computed as a function of the following discovery trait parameters: (1) the discovery sample size set based on the maximum sample size from the multivariate analyses, excluding Netherlands Twin Register Participants (*N* = 2,281,978) and (2) the discovery trait SNP heritability (*h*_snp_), as estimated by Baselmans et al. (2019) in the target sample, set at 0.02. Concerning the target trait parameters, we adjusted the parameters according to the different phenotypes mentioned above and set the significance threshold at a Bonferroni corrected value of α = 0.005/11 = 0.0005, where 11 represents the number of phenotypes to be predicted with the polygenic scores. Table 2 shows an overview of the different input parameters and the results of the power analyses. The estimated SNP heritability for personality, self-rated health, loneliness, and depressive symptoms was based on results from previous studies (Okbay et al. 2016; Harris et al. 2016; Lo et al. 2017; Gao et al. 2017). The SNP heritability for the 3-WBS was estimated using LD score regression (Bulik-Sullivan et al. 2015). Since there has been no genome-wide association study for flourishing, we estimated the SNP heritability to be approximately as high as the SNP heritability for subjective well-being and meaning in life, which are estimated at ~ 0.04 (Okbay et al. 2016) and ~ 0.06 (Baselmans and Bartels 2018), respectively. The power for all traits was very high, assuming a medium to high genetic correlation, with the exception of flourishing, where (due to smaller sample and low SNP heritability) the power to detect effects was somewhat lower, around 0.60 (assuming a genetic correlation of ~ 0.8).

**Table 2:** Power calculation parameters for the polygenic prediction

Discovery trait parameters						
Well-being spectrum	Nobs	SNP heritability				
	2,281,978	0.021				

Target trait parameters	Input sample size	SNP heritability	Power if r_g_ = 0.2	Power if r_g_ = 0.4	Power if r_g_ = 0.6	Power if r_g_ = 0.8
Subjective well-being	5300	0.04	0.07	0.46	0.90	0.99
Neuroticism	8600	0.12	0.56	0.99	1	1
Depressive symptoms	8600	0.05	0.18	0.70	0.99	1
Loneliness	8800	0.16	0.74	1	1	1
Openness to experience	8600	0.11	0.51	0.99	1	1
Conscientiousness	8600	0.10	0.45	0.99	1	1
Extraversion	8600	0.18	0.79	1	1	1
Agreeableness	8600	0.09	0.4	0.99	1	1
Self-rated health	8600	0.13	0.61	0.99	1	1
Flourishing	2200	0.04	0.02	0.15	0.44	0.77

The power was estimated based on alpha = 0.005. r_g_ = genetic correlation

### Polygenic prediction

The polygenic scores were created using LDpred (Vilhjálmsson et al. 2015). LDpred takes into account linkage disequilibrium (LD) among SNPs in creating the polygenic risk scores. We calculated the mean causal effect size of each marker using the SNP effect sizes from the recent multivariate 3-WBS GWAMA, where SNP effects were reversed for depressive symptoms and neuroticism, ensuring that a higher score reflects higher levels of well-being (Baselmans et al. 2019). The LD structure from a reference set specific for the NTR based on 1000 Genomes phase 1 genotypes (1000 Genomes Project Consortium, 2015) was used to calculate polygenic scores in the target sample. In order to avoid an over-estimation of the association between the polygenic scores and phenotypes, summary statistics in the discovery set were re-computed, excluding NTR subjects (resulting in *N* = 2,281,978 observations). Polygenic scores were calculated with the fractions of causal genetic variants (the fraction of markers with non-zero effects) set to 1, 0.3, 0.1, 0.03, 0.01 to test which fraction suited the data best. Generalized Estimating Equation (GEE) modelling was used to test whether the 3-WBS polygenic scores significantly predict satisfaction with life, happiness, neuroticism, depressive symptoms, loneliness, openness to experience, conscientiousness, extraversion, agreeableness, self-rated health, and flourishing. An exchangeable conditional covariance matrix was used to account for family relatedness and tests were based on robust (sandwich-corrected) standard errors (Minica et al. 2014). Age, age^2^, sex, and the first ten genomic principal components (PCs) (three ancestry-informative PCs and seven PCs accounting for genotyping batch effects) were included as covariates. To obtain 95% confidence intervals (CI) around the R^2^’s, we used the R-package Psychometrics (Schwarzer 2007).

### Genetic correlations

We used LD score regression (Bulik-Sullivan et al. 2015) to compute the genetic correlations between the 3-WBS and the candidate traits for which genome-wide association study (GWAS) summary statistics were available. This method distinguishes bias and inflation from a true polygenic signal by quantifying the contribution of each through examining the relationship between linkage disequilibrium and test statistics. For neuroticism, depressive symptoms, positive affect, and life satisfaction, we used the univariate summary statistics from the multivariate 3-WBS GWAMA (Baselmans et al. 2019). For all personality measures except neuroticism, we used summary statistics from a subset of 23 and me participants (Lo et al. 2017).

We obtained summary statistics for self-rated health and loneliness by running GWASs on data from UK Biobank (UK Biobank ID 20459 and 2020 under UK Biobank approval 25472, respectively). Genome-wide association analyses were performed in PLINK (Purcell et al. 2007) in a linear regression model of additive allelic effects. Standard pre-GWAS quality control filters were applied, which included removing SNPs with minor allele frequency < 0.005 and/or with an INFO-score < 0.8 for imputed SNPs, and removing individuals with ambiguous sex and/or non-British ancestry. Furthermore, we randomly selected 1 individual from each closely related pair of relatives (i.e. parent offspring pairs, sibling pairs). The GWAS included 40 principal components, age, sex, and a chip dummy as covariates. The summary statistics from these GWASs were used as input for LD score regression analyses. More detailed information on the different GWASs used as input for the genetic correlation analysis can be found in the Online Resource 2. The significance threshold for the genetic correlations was set at a Bonferonni corrected value of α = 0.005/55 = 0.00009.

### Factor analysis in genomic SEM

We performed exploratory and confirmatory factor analysis using the r-package Genomic SEM (Grotzinger et al. 2018). This method performs structural equation modelling using GWA summary statistics, allowing us to explore the genetic factor structure of the WBS traits. To perform the analyses in approximately independent datasets, we first split all summary statistics files in one containing the even chromosomes, and one containing the odd chromosomes.

Exploratory factor analysis (EFA) was performed with the summary statistics on the even chromosomes only. We first conducted EFA with promax rotation and 1, 2 or 3 factors on all nine traits for which we had summary statistics available. Based on this analysis, we excluded traits that clearly fall outside of the spectrum because their factor loadings were zero, or close to zero. The remaining traits were subjected to another round of EFA. Next, we performed confirmatory factor analysis (CFA) on the odd chromosomes to assess what factor structure had the best model fit.

## Results

### Phenotypic correlations

**Fig. 1:**
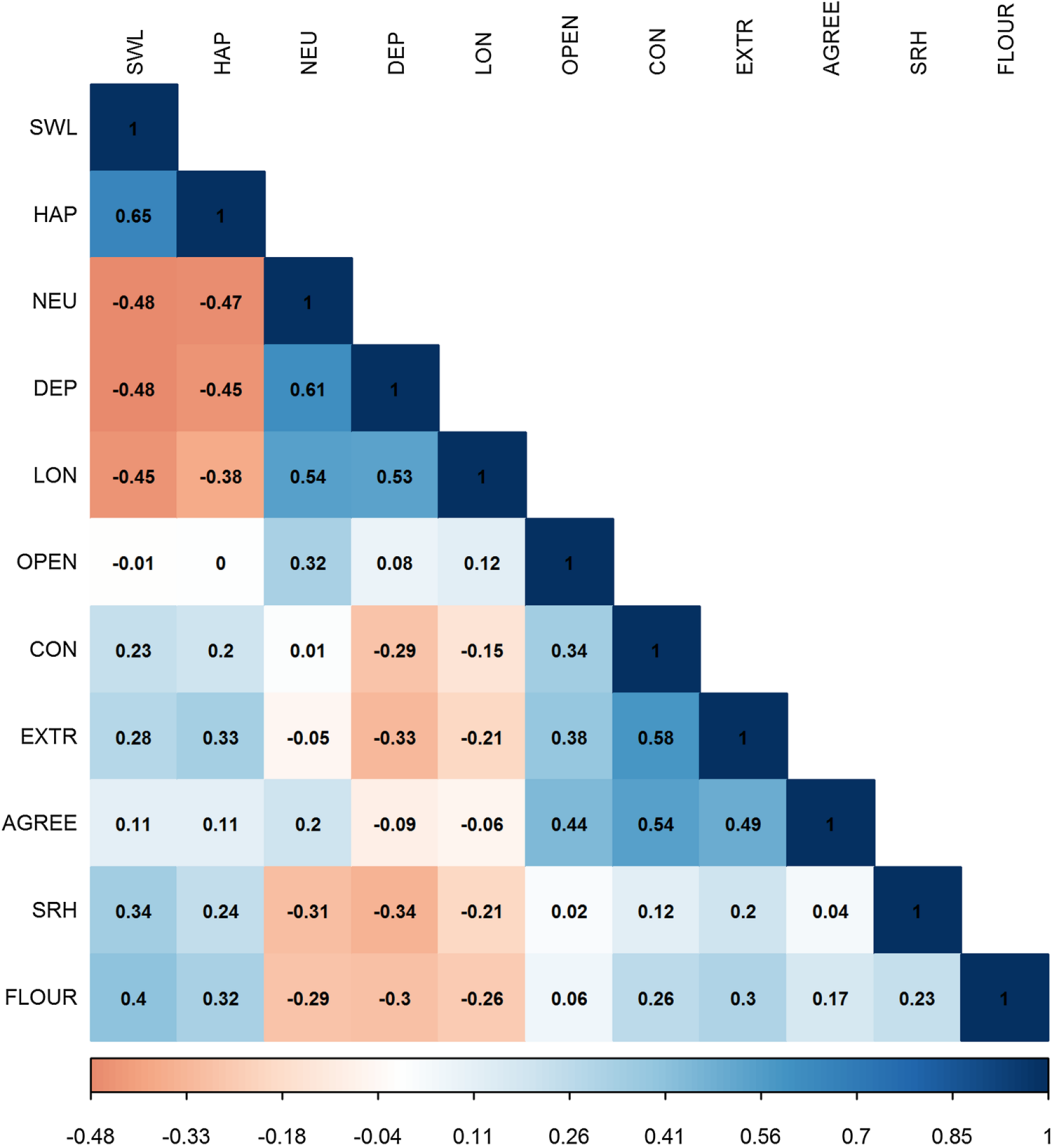
Phenotypic correlations between the different traits. SWL = satisfaction with life, HAP = happiness, NEU = neuroticism, DEP = depressive symptoms, LON = loneliness, OPEN = openness to experience, CON = conscientiousness, EXTR = extraversion, AGREE = agreeableness, SRH = self-rated health, FLOUR = flourishing

Figure 1 and Online Resource 3 show the phenotypic correlation structure between the traits as measured in the NTR. The correlations depicted in red and blue depict negative and positive associations, respectively. The well-being phenotypes satisfaction with life and happiness were significantly associated with all traits except openness to experience. Neuroticism was associated with all traits except conscientiousness. All traits were significantly correlated with depressive symptoms. The 3-WBS traits were most significantly associated with each other, followed by the correlations between the 3-WBS phenotypes and loneliness (weakest *r* = − 0.38 and strongest *r* = 0.53), self-rated health (weakest *r* = − 0.31 and strongest *r* = 0.41), and flourishing (weakest *r* = − 0.29 and strongest *r* = 0.40).

### Polygenic prediction

**Fig. 2:**
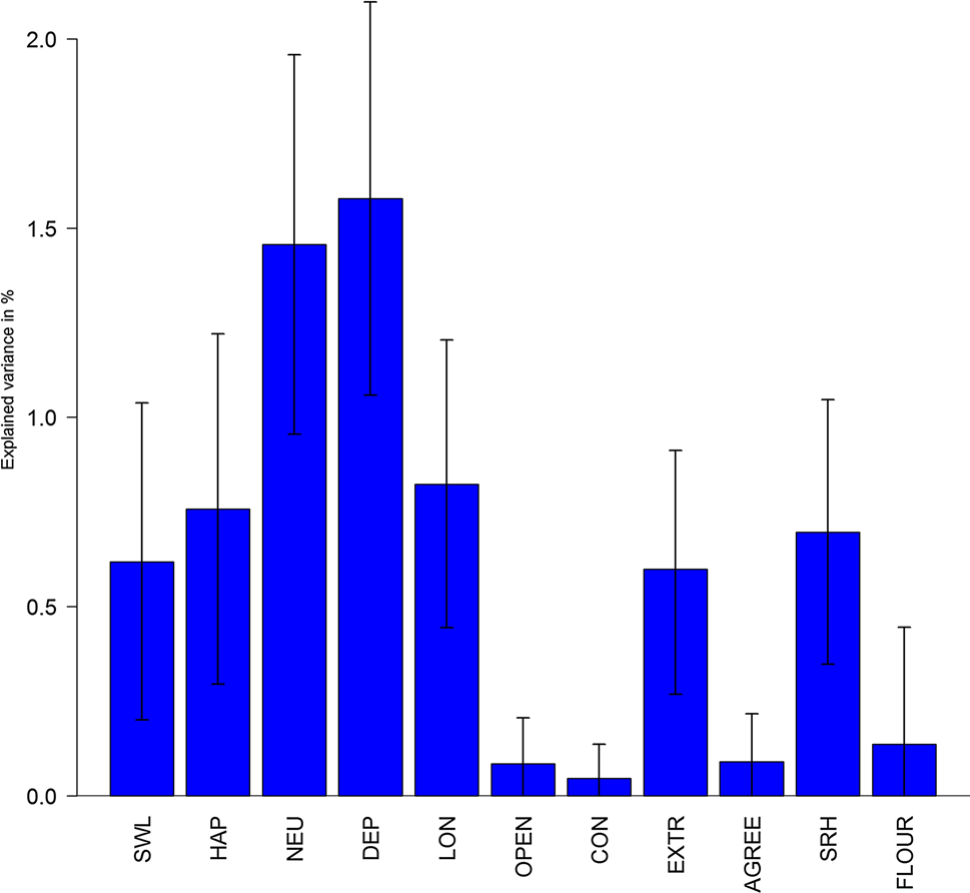
The amount of variance explained by the 3-WBS polygenic risk score for each of the traits. SWL = satisfaction with life, HAP = happiness, NEU = neuroticism, DEP = depressive symptoms, LON = loneliness, OPEN = openness to experience, CON = conscientiousness, EXTR = extraversion, AGREE = agreeableness, SRH = self-rated health, FLOUR = flourishing

Online Resource 4 shows the results of the Polygenic Scores using five different fractions. We found that polygenic scores using a fraction of 1 show the best prediction results. Figure 2 shows the results from the GEE analyses where the polygenic scores for the 3-WBS were used to predict the eleven outcome variables. As a proof of principle, we found that the traits used to create the polygenic scores (satisfaction with life, happiness, neuroticism, and depressive symptoms) were significantly associated with the polygenic score (Online Resource 5). From the candidate traits to be added to a well-being spectrum, three were significantly associated with the 3-WBS polygenic score. The strongest association was found for loneliness (R^2^ = 0.82), followed by self-rated health (R^2^ = 0.70), and extraversion (R^2^ = 0.60). Conscientiousness, agreeableness, and flourishing were not significantly predicted by the 3-WBS polygenic score.

### Genetic correlations

**Fig. 3:**
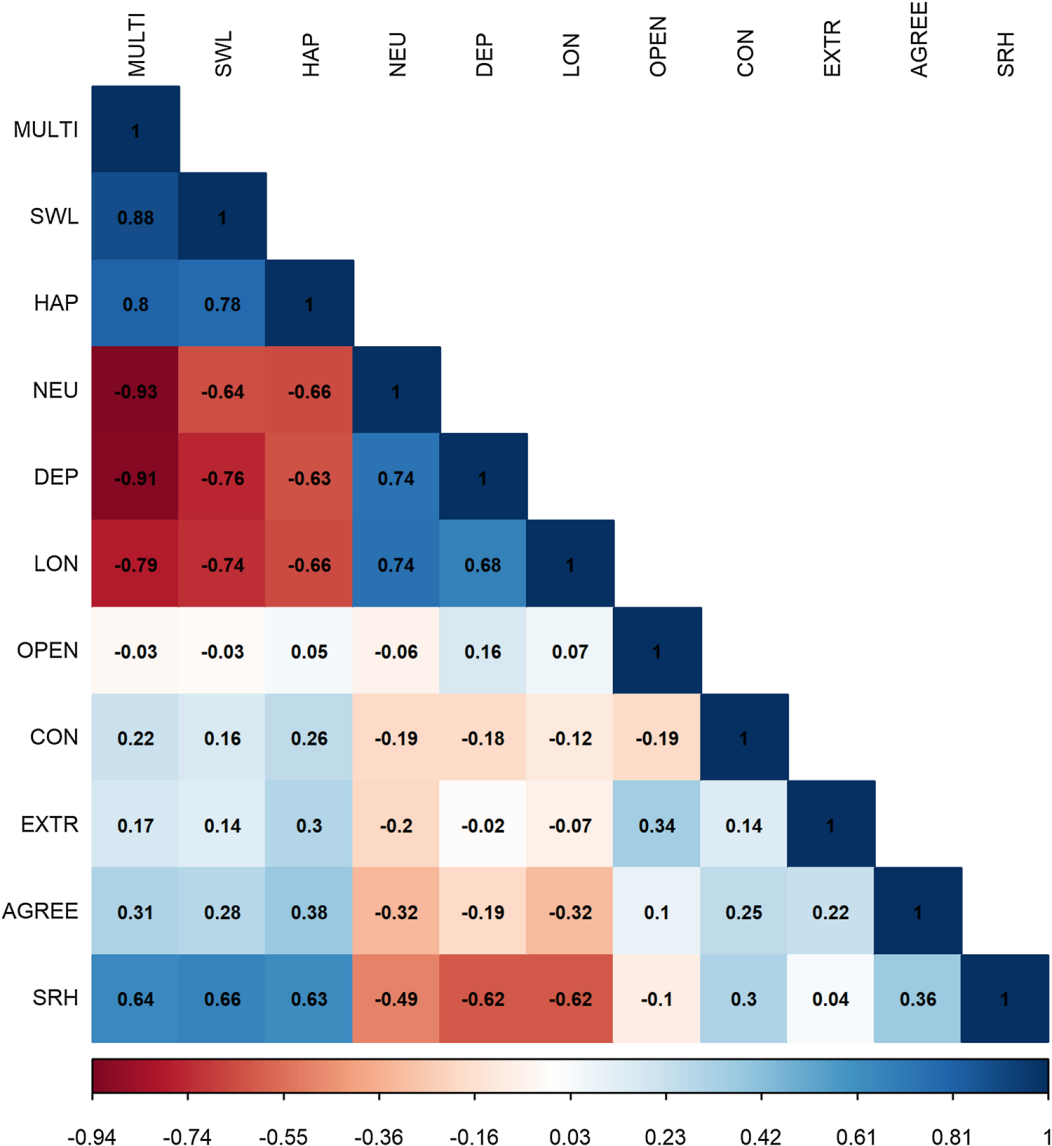
Genetic correlations between the different traits. MULTI = multivariate 3-WBS, SWL = satisfaction with life, HAP = happiness, NEU = neuroticism, DEP = depressive symptoms, LON = loneliness, OPEN = openness to experience, CON = conscientiousness, EXTR = extraversion, AGREE = agreeableness, SRH = self-rated health

Figure 3 and Online Resource 6 depict the genetic correlations obtained using LD score regression. Again, the correlations depicted in red and blue depict negative and positive associations, respectively. As expected, the genetic correlations were strongest between the 3-WBS and the traits originally included in the spectrum, life satisfaction (*r*_g_ = 0.88), positive affect (*r*_g_ = 0.80), neuroticism (*r*_g_ = − 0.93), and depressive symptoms (*r*_g_ = − 0.91). Next, loneliness had the strongest genetic correlation with 3-WBS (*r*_g_ = − 0.79), followed by self-rated health (*r*_g_ = 0.64), agreeableness (*r*_g_ = 0.31), conscientiousness (*r*_g_ = 0.22), and extraversion (*r*_g_ = 0.17). The only trait that did not have a significant genetic association with 3-WBS was openness to experience (*r*_g_ = − 0.03). As a negative control we used height, which showed no significant genetic correlation with any of the phenotypes (see Online Resource 7).

### Genomic SEM

The genetic correlation and polygenic score analyses revealed that the original 3-WBS traits are most strongly genetically correlated with loneliness and SRH. To further provide evidence for their relatedness, we applied Genomic SEM. First, an EFA on all nine traits was conducted. This analysis, in accordance with the previous results, showed that agreeableness, conscientiousness, extraversion, and openness to experience poorly behaved in the proposed factor-structures (zero to very low factor loadings, see Online Resource 8). As EFA with all traits resulted in poor factor loadings, we repeated EFA with only the original 3-WBS traits and loneliness and SRH, with 1 and 2 factors and promax rotation. The factor loadings can be found in Online Resource 9. The EFA pattern of clustering in the two-factor model suggested one factor with depressive symptoms, neuroticism and loneliness, and a second consisting of life satisfaction, positive affect and self-rated health.

**Fig. 4:**
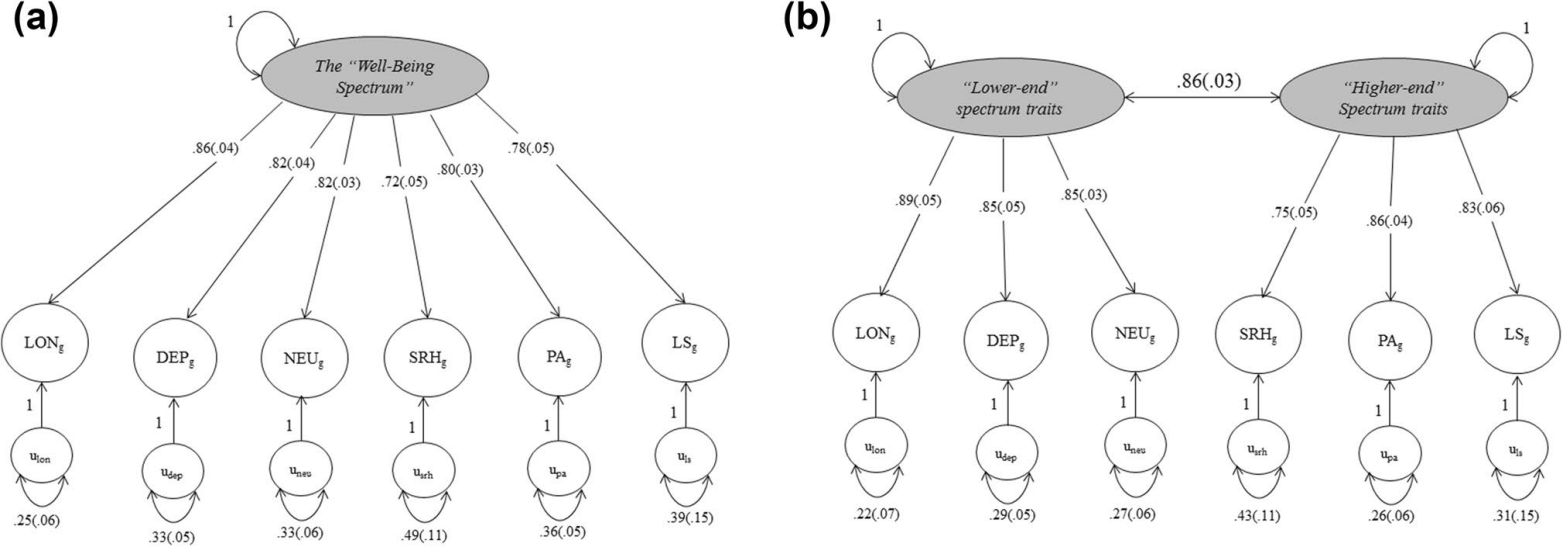
Path models Genomic SEM CFA with **a** one and **b** two factors

We evaluated the one- and two- factor model fit using CFA. The results are shown in Fig. 4. While the goodness-of-fit indices (Table 3) supported a reasonably good fit for both the one- (*X*^*2*^ = 51,257, *df* = 9) and two-factor model (*X*^*2*^ = 26,116, *df* = 8), the two-factor model had a slightly better fit. In this two-factor model, the correlation between the two latent factors was very high (0.86). To confirm this high latent factor correlation, we also ran a model where the latent factors were not allowed to correlate. Without a latent factor correlation, the model fit decreased dramatically (*X*^*2*^ = 1167,503, *df* = 9), confirming the high relatedness between the two latent factors.

**Table 3:** Fit indices confirmatory factor analysis

Model	χ^2^	*df*	AIC	CFI	SRMR
1 Factor	51,257	9	75,257	0.982	0.058
2 Correlated factors	26,116	8	52,116	0.992	0.040
2 Uncorrelated factors	1167,503	9	1691,503	0.282	0.399

*Df* degrees of freedom, *AIC* Akaike Information Criterion, *CFI* Comparative Fit Index, *SRMR* standardized root mean residual

## Discussion

Well-being is a broad construct, with many traits contributing to its variation. In this study, we applied three types of genetic analyses to examine the genetic boundaries of a well-being spectrum: polygenic score prediction, genetic correlation analyses, and genomic structural equation modelling. The traits we examined included the original proposed 3-WBS (subjective well-being, depressive symptoms & neuroticism), as well as loneliness, openness to experience, conscientiousness, extraversion, agreeableness, self-rated health, and flourishing. The results supported the inclusion of loneliness and self-rated health in an extended 5-WBS, where depression, neuroticism, and loneliness cluster at the “negative-end” of the spectrum, and satisfaction with life, happiness and self-rated health cluster at the “positive-end”.

Importantly, the traits originally proposed for the WBS by Baselmans et al. (2019) were validated in this study: both the results from the polygenic score prediction and genetic correlation analyses were in line with strong genetic associations between these traits. This also confirms previous findings that indicate high genetic correlations between life satisfaction, positive affect, neuroticism, and depressive symptoms (Okbay et al. 2016). Polygenic score prediction and genetic correlation analyses of the candidate traits provided a first indication of the potential extension of the WBS with loneliness and self-rated health. The PRS significantly predicted loneliness, self-rated health and extraversion, and we found significant genetic correlations with loneliness, self-rated health, agreeableness, conscientiousness, and extraversion. While based on these results one could conclude that there are more traits that could potentially be included in the spectrum, we strongly believe that the genetic overlap was only of significant magnitude between the original 3-WBS and loneliness and self-rated health.

To confirm this, and to further investigate the genetic structure of our hypothesized spectrum, we performed genomic structural equation modelling. EFA supported the inclusion of only our proposed 5-WBS traits for CFA. The model that best fit the data was a two-factor model with two highly correlated factors, with one latent factor represented by the “negative-end of the spectrum” traits depression, neuroticism and loneliness, and the other factor the “positive-end of the spectrum” traits satisfaction with life, happiness and self-rated health. The high correlation between the two factors suggests the existence of a potential higher-order factor, but with only two sub-factors, it was not statistically sensible to test for this model. Possibly, future investigations of the proposed traits that further subdivide the traits (e.g. satisfaction with financial situation, satisfaction with health, etc.) could shed more light on this potential higher-order factor. While we initially expected to find a one-factor model for this extended WBS, the two factor model also supports the theoretical structure of the spectrum, given the high correlation between the two factors and the clustering of negative-end and positive-end traits.

Surprisingly, we found no evidence for the inclusion of flourishing in our proposed spectrum. Since the flourishing scale is a measure of PWB, and PWB is phenotypically highly associated with subjective well-being (Nave et al. 2008; Vanhoutte and Nazroo 2014), we expected that, in line with the recent work of Baselmans and Bartels (Baselmans and Bartels 2018), part of this association could be explained by genetic factors. Two explanations are possible for our observations. The first explanation is that the relationship between 3-WBS and PWB as defined in this study is mainly a result of environmental factors. The second explanation is that, since our study had relatively low power to detect associations for flourishing, there is genetic overlap, but that these genetic effects remained unnoticed in this study. Unfortunately, we could not calculate the genetic correlation between the 3-WBS and flourishing due to the constraint of the absence of a genome-wide association summary statistics for flourishing. However, future studies with larger sample sizes for PWB measures could elucidate which of these explanations is correct.

We note that the genetic correlations suggest large genetic overlap between the several traits, whereas the polygenic risk scores only explain a small part of the variance in each trait even with our large discovery sample. This discrepancy can be expected since the genotyped SNPs do not necessarily tag all causal variants, and not all SNPs were genotyped. Moreover, since measurement error accumulates across all the markers, sampling variation has a large influence on the predictive accuracy of the polygenic score (Dudbridge 2013).We are therefore optimistic that, with increasing sample sizes and increased accuracy in the estimation of SNP effects, well-being polygenic scores will gain substantial predictive power.

The presented results provide us with useful information on the determinants of individual differences in human well-being. Even though not all traits examined here can be included in the well-being spectrum from a genetic point of view, most of them are phenotypically and/or genetically related to well-being to some extent. It is important to identify such correlates, since it could help us improve policy making and clinical interventions aimed at improving human well-being. However, the results should be interpreted with caution. As shown in Table 2, the power of the polygenic prediction is dependent on sample size, especially when the genetic correlation between traits is low. Thus, better predictive accuracy and power could be achieved with larger sample sizes. Moreover, while including more traits in the well-being spectrum can lead to greater power for detecting genetic variants, the number of genetic variants influencing all traits will decline. While an increase in power when using the multivariate 3-WBS as opposed to univariate analyses in genome-wide analyses has been validated by Baselmans et al. (2019), we have not yet validated an increase in power when using all 5-WBS traits. Here we describe the first evidence for an extended spectrum, but an extensive multivariate genome-wide meta-analysis is beyond the scope of the this paper. Future studies taking a multivariate approach are therefore recommended.

To conclude, in this study we confirm a shared genetic aetiology between several traits associated with well-being using multiple genetic methods. The strongest relationships were found for loneliness and self-rated health. Our findings suggest that these two traits should be further investigated for potential inclusion in the well-being spectrum to increase our understanding of the causes and links between well-being and several mental/behavioural traits.

## Electronic supplementary material

Below is the link to the electronic supplementary material.

Supplementary material 1 (DOCX 454 KB)
