# Supplementary material for: A Genetic Investigation of the Well-Being Spectrum
Source: Behav Genet. 2019 Feb 27;49(3):286–97. doi: 10.1007/s10519-019-09951-0 (PMC6497622; doi:10.1007/s10519-019-09951-0)
Supplement: Supplementary file 1 — Supplementary material 1 (DOCX 454 KB) [file 10519_2019_9951_MOESM1_ESM.docx]

**Online Supplementary Materials**Online Resource 1.
*Histograms of the distribution of phenotypes within the Netherlands Twin Register.*


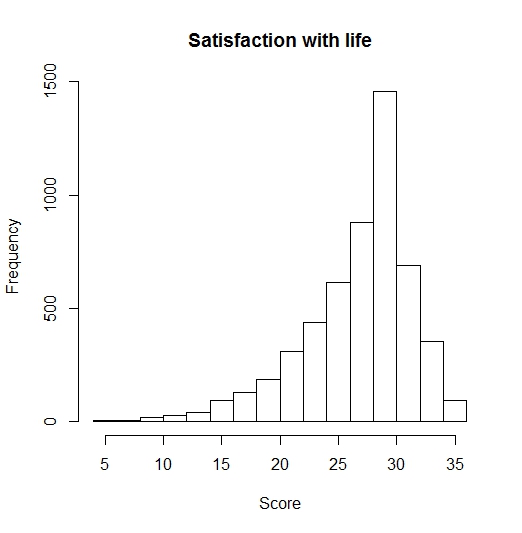

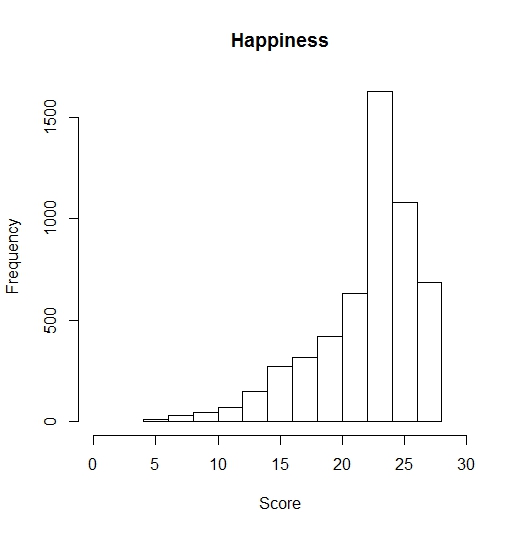

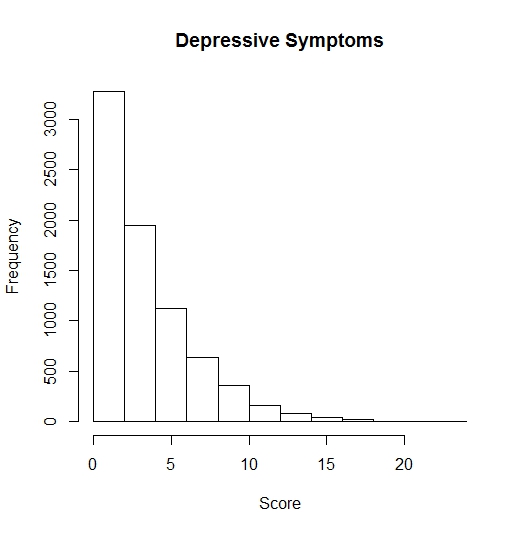

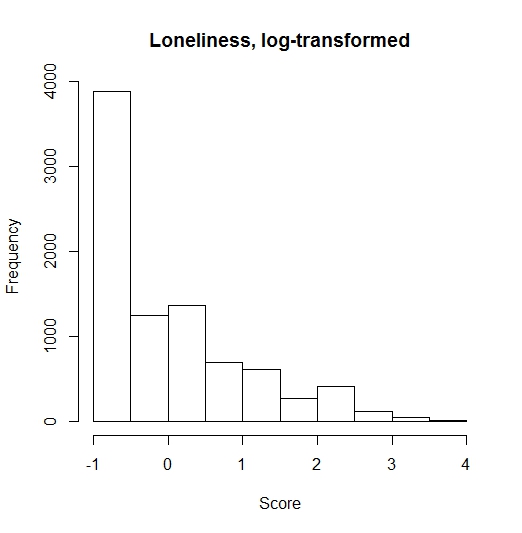


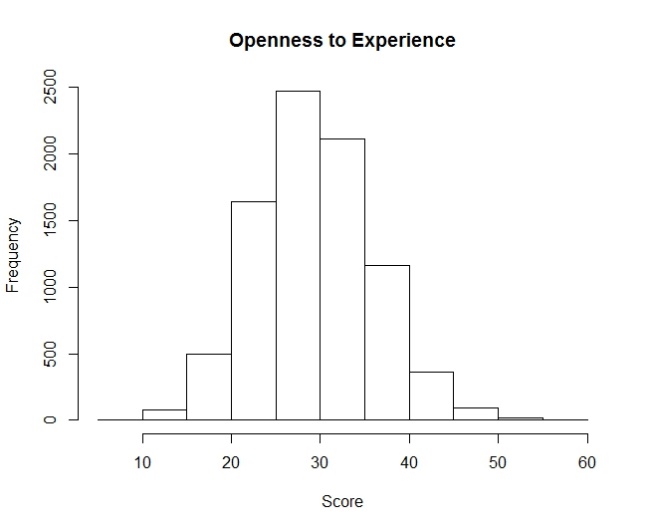

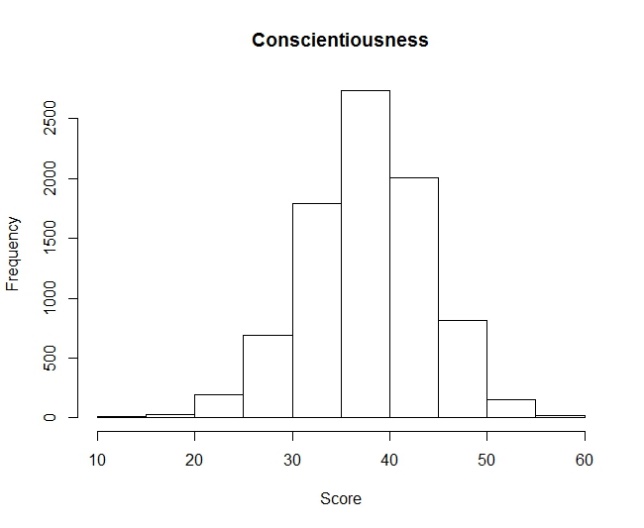


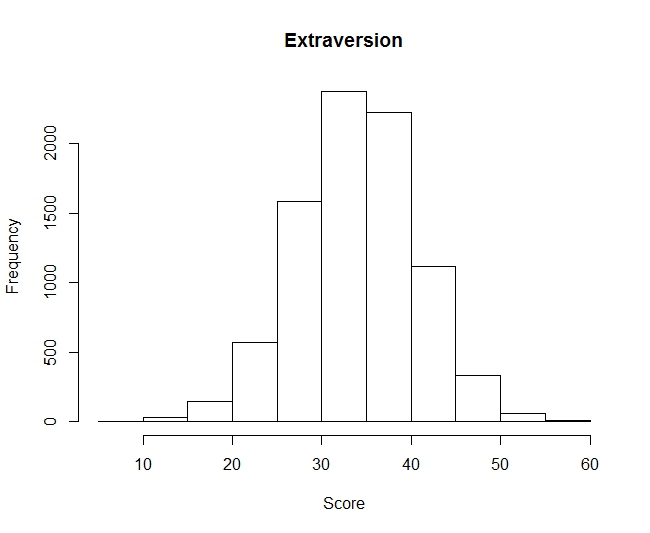

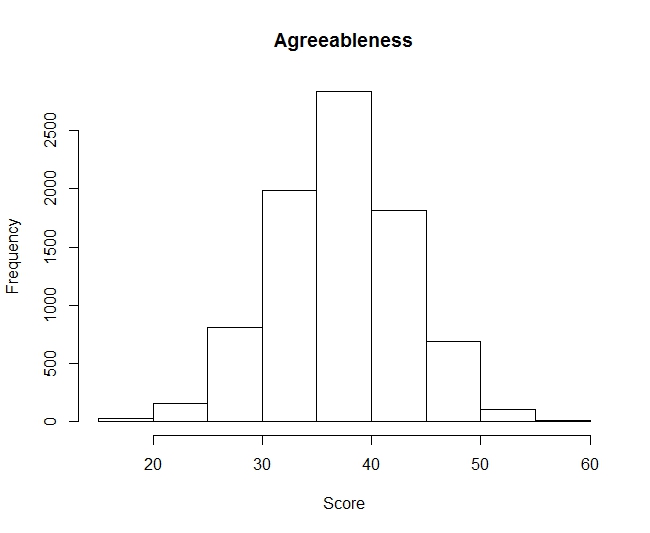


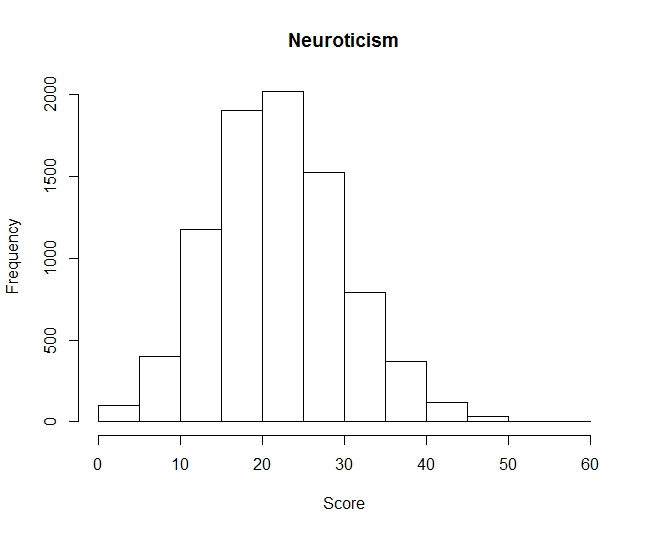

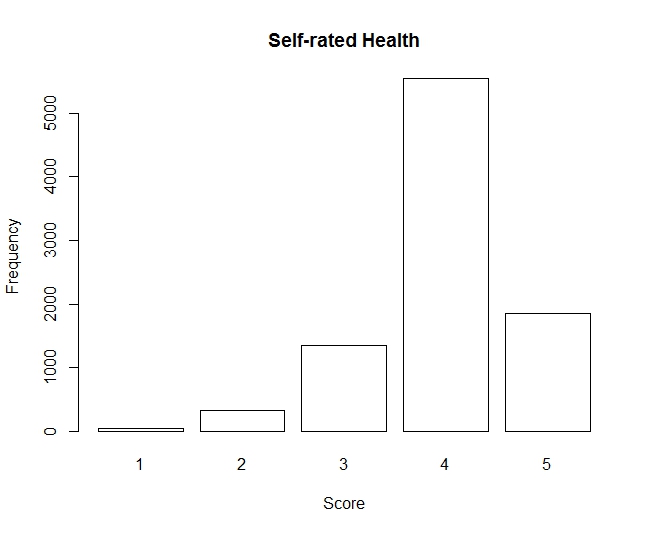


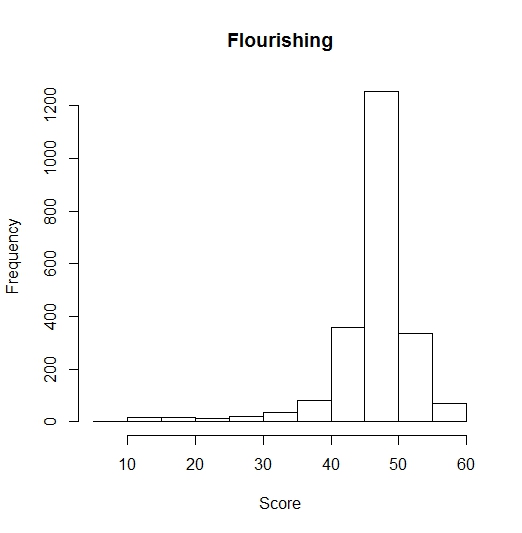


| Online Resource 2  Descriptives Samples | |  |  |  | |
| --- | --- | --- | --- | --- | --- |
| Trait | Sample(s) | *N* | *h^2^_snp_** | Ancestry |  |
| 3-WBS | 23andme, CHARGE, UKB, SSGAC, US | 2,370,390 | ~0.02 | European | |
| Satisfaction with Life | UKB, SSGAC, US | 80,852 | ~0.03 | European | |
| Positive Affect | SSGAC,US | 410,603 | ~0.04 | European | |
| Neuroticism | UKB, 23andme, SSGAC, US | 582,989 | ~0.08 | European | |
| Depressive Symptoms | 23andme CHARGE UKB, SSGAC, US | 1,295,946 | ~0.03 | European | |
| Loneliness | UKB | #246,787 | ~0.06 | European | |
| Openness to Experience | 23andme | 76,581 | ~0.08 | European | |
| Conscientiousness | 23andme | 76,551 | ~0.07 | European | |
| Extraversion | 23andme | 122,886 | ~0.09 | European | |
| Agreeableness | 23andme | 76,551 | ~0.07 | European | |
| Self-Rated Health | UKB | 110,411 | ~0.07 | European | |
|  | |  |  |  | |
| *The SNP heritability reflects the heritability as estimated by LD Score Regression in our analyses.  # Effective sample size | | | | | |

|  | **SWL** | **HAP** | **NEU** | **DEP** | **LON** | **OPEN** | **CON** | **EXTR** | **AGREE** | **SRH** | **FLOUR** |
| --- | --- | --- | --- | --- | --- | --- | --- | --- | --- | --- | --- |
| **SWL** | 1 | - | - | - | - | - | - | - | - | - | - |
| **HAP** | .647(.014)* | 1 | - | - | - | - | - | - | - | - | - |
| **NEU** | -.478(.014)* | -.465(.015)* | 1 | - | - | - | - | - | - | - | - |
| **DEP** | -.482(.020)* | -.446(.019)* | .599(.010)* | 1 | - | - | - | - | - | - | - |
| **LON** | -.445(.016)* | -.382(.016)* | .533(.010)* | .543(.014)* | 1 | - | - | - | - | - | - |
| **OPEN** | -.016(.015) | .001(.015) | .313(.012)* | .081(.013)* | .113(.012)* | 1 | - | - | - | - | - |
| **CON** | .226(.016)* | .202(.015)* | .011(.012) | -.295(.014) | -.146(.012)* | .337(.115)* | 1 | - | - | - | - |
| **EXTR** | .277(.016)* | .333(.015)* | -.065(.012)* | -.335(.014)* | -.204(.013)* | .379(.011)* | .578(.010)* | 1 | - | - | - |
| **AGREE** | .111(.016)* | .114(.015)* | .192(.012)* | -.088(.013)* | -.060(.012)* | .439(.011)* | .540(.010)* | .490(.011)* | 1 | - | - |
| **SRH** | .399(.015)* | .305(.015)* | -.308(.012)* | -.407(.014)* | -.228(.013)* | -.036(.012) | .138(.012)* | .223(.012)* | .036(.012) | 1 | - |
| **FLOUR** | .397(.045)* | .315(.039)* | -.287(.034)* | -.302(.038)* | -.266(.032)* | .060(.025) | .263(.029)* | .301(.030)* | .166(.023)* | .242(.025)* | 1 |

Online Resource 3.

*Phenotypic Correlations Between the Traits (NTR Data).*

*Note*. SWL= Satisfaction with Life, HAP = Happiness, NEU= Neuroticism, DEP= Depressive Symptoms, LON= Loneliness, OPEN= Openness to Experience , CON= Conscientiousness, EXTR = Extraversion, AGREE= Agreeableness, SRH= Self-Rated Health, FLOUR= Flourishing.

*p-value significant at α=.00009 (0.005/55).6

Online Resource 4

Polygenic Scores

R-square calculated using five different fractions (1, 0.3, 0.1, 0.03, 0.01) for loneliness, Neuroticism, Extraversion, Openness, Agreeableness, Conscientiousness, Satisfaction with Life, Happiness, Depression, Self-Rated Health, Flourishing. R-square calculated with fraction 0.3 provides the best prediction results and will be used.

|  | fractions |  |  |  |  |
| --- | --- | --- | --- | --- | --- |
| Phenotypes | 1 | 0.3 | 0.1 | 0.03 | 0.01 |
| SWL | **0.619** | 0.535 | 0.499 | 0.227 | 0.009 |
| HAP | **0.758** | 0.661 | 0.530 | 0.166 | 0.007 |
| NEU | **1.457** | 1.452 | 0.942 | 0.658 | 0.134 |
| DEP | **1.578** | 1.522 | 1.016 | 0.530 | 0.156 |
| LON | **0.824** | 0.807 | 0.546 | 0.275 | 0.061 |
| OPEN | **0.084** | 0.073 | 0.103 | 0.071 | 0.012 |
| CON | **0.046** | 0.034 | 0.026 | 0.014 | 0.011 |
| EXTR | **0.599** | 0.540 | 0.288 | 0.128 | 0.076 |
| AGREE | **0.090** | 0.096 | 0.021 | 0.007 | 0.007 |
| SRH | **0.697** | 0.631 | 0.366 | 0.151 | 0.031 |
| FLOUR | **0.136** | 0.091 | 0.173 | 0.057 | 0.012 |

*Note*. SWL= Satisfaction with Life, HAP = Happiness, NEU= Neuroticism, DEP= Depressive Symptoms, LON= Loneliness, OPEN= Openness to Experience , CON= Conscientiousness, EXTR = Extraversion, AGREE= Agreeableness, SRH= Self-Rated Health, FLOUR= Flourishing.

Online Resource 5

Polygenic Scores

Outcome GEE analyses for loneliness, Neuroticism, Extraversion, Openness, Agreeableness, Conscientiousness, Satisfaction with Life, Happiness, Depression, Self-Rated Health, Flourishing. The three columns at the right represent the R2 in percentage and their corresponding 95% confident intervals.

|  | **Estimate** | **Naïve SE** | **Naïve Z** | **Robust SE** | **Robust Z** | **P** |  | **R2 (%)** | **R2 95% low** | **R2 95% high** |
| --- | --- | --- | --- | --- | --- | --- | --- | --- | --- | --- |
| SWL | 0.079 | 0.014 | 5.771 | 0.015 | 5.302 | 1.14E-07 |  | 0.619 | 0.200044 | 1.037956 |
| HAP | 0.087 | 0.014 | 6.378 | 0.015 | 5.926 | 3.09E-09 |  | 0.758 | 0.295292 | 1.220708 |
| NEU | -0.121 | 0.011 | -11.372 | 0.012 | -10.026 | 1.17E-23 |  | 1.457 | 0.955087 | 1.958913 |
| DEP | -0.126 | 0.011 | -11.354 | 0.012 | -10.427 | 1.87E-25 |  | 1.578 | 1.057658 | 2.098342 |
| LON | -0.091 | 0.011 | -8.560 | 0.011 | -8.082 | 6.39E-16 |  | 0.824 | 0.444123 | 1.203877 |
| OPEN | -0.029 | 0.011 | -2.682 | 0.012 | -2.330 | 1.98E-02 |  | 0.084 | -0.03819 | 0.206193 |
| CON | 0.021 | 0.011 | 1.995 | 0.012 | 1.780 | 7.50E-02 |  | 0.046 | -0.04446 | 0.136459 |
| EXTR | 0.077 | 0.011 | 7.154 | 0.012 | 6.450 | 1.12E-10 |  | 0.599 | 0.267798 | 0.912202 |
| AGREE | 0.030 | 0.010 | 2.878 | 0.012 | 2.577 | 9.98E-03 |  | 0.09 | -0.03647 | 0.216475 |
| SRH | 0.084 | 0.010 | 8.264 | 0.011 | 7.887 | 3.10E-15 |  | 0.697 | 0.348083 | 1.045917 |
| FLOUR | 0.037 | 0.021 | 1.725 | 0.022 | 1.693 | 0.090460457 |  | 0.136 | -0.17135 | 0.445349 |

*Note*. SWL= Satisfaction with Life, HAP = Happiness, NEU= Neuroticism, DEP= Depressive Symptoms, LON= Loneliness, OPEN= Openness to Experience , CON= Conscientiousness, EXTR = Extraversion, AGREE= Agreeableness, SRH= Self-Rated Health, FLOUR= Flourishing.

*p-value significant at α=.0005.

Online Resource 6

Genetic Correlations.

|  | **3WBS** | **SWL** | **PA** | **NEU** | **DEP** | **LON** | **OPEN** | **CON** | **EXTR** | **AGREE** | **SRH** |
| --- | --- | --- | --- | --- | --- | --- | --- | --- | --- | --- | --- |
| **3WBS** | 1 | - | - | - | - | - | - | - | - | - | - |
| **SWL** | .880(.083)* | 1 | - | - | - | - | - | - | - | - | - |
| **PA** | .801(.014)* | .777(.051)* | 1 | - | - | - | - | - | - | - | - |
| **NEU** | -.929(.005)* | -.645(.057)* | -.660(.018)* | 1 | - | - | - | - | - | - | - |
| **DEP** | -.912(.006)* | -.759(.085)* | -.631(.023)* | .738(.015)* | 1 | - | - | - | - | - | - |
| **LON** | -.787(.018)* | -.735(.068)* | -.658(.026)* | .740(.017)* | .678(.022)* | 1 | - | - | - | - | - |
| **OPEN** | -.031(.047) | -.029(.065) | .046(.043) | -.062(.048) | .162(.042) | .069(.051) | 1 | - | - | - | - |
| **CON** | .215(.041)* | .164(.068) | .258(.045) | -.194(.038)* | -.183(.043)* | -.117(.048) | -.187(.064) | 1 | - | - | - |
| **EXTR** | .174(.037)* | .137(.055) | .296(.034)* | -.204(.037)* | -.018(.036) | -.074(.038) | .341(.045)* | .145(.053) | 1 | - | - |
| **AGREE** | .309(.046)* | .284(.072)* | .375(.046)* | -.322(.047)* | -.192(.042)* | -.320(.055)* | .095(.073) | .252(.065) | .221(.052) | 1 | - |
| **SRH** | .640(.038)* | .663(.076)* | .629(.034)* | -.492(.046)* | -.617(.038)* | -.616(.044)* | -.098(.056) | .305(.054)* | .039(.047) | .036(.063) | 1 |

*Genetic Correlations (SE) Between the Different Traits (Data From Several GWAS).*

*Note.* 3WBS=3-Trait Well-Being Spectrum, SWL= Satisfaction with Life, PA= Positive Affect, NEU= Neuroticism, DEP= Depressive Symptoms, LON= Loneliness, OPEN= Openness to Experience, CON= Conscientiousness, EXTR= Extraversion, AGREE= Agreeableness, SRH= Self-Rated Health.
*p-value significant at *α*= .00009 (0.005/55).

Online Resource 7. Genetic Correlation with Height as a negative control

| **pheno 1** | **pheno 2** | | **rg** | | **se** | | **z** | | **p** | |
| --- | --- | --- | --- | --- | --- | --- | --- | --- | --- | --- |
| SWB (multi) | Height | -0.0491 | | 0.0169 | | -2.897 | | 3.77E-03 | |  |
| DEP | Height | -0.0537 | | 0.0182 | | -2.9478 | | 3.20E-03 | |  |
| EXT | Height | 0.0004 | | 0.0233 | | 0.019 | | 9.85E-01 | |  |
| NEU | Height | -0.0603 | | 0.0184 | | -3.273 | | 1.06E-03 | |  |
| LON | Height | -0.0789 | | 0.0248 | | -3.1844 | | 1.45E-03 | |  |
| SRH | Height | -0.0009 | | 0.0291 | | -0.031 | | 9.75E-01 | |  |
| PA | Height | -0.003 | | 0.0226 | | -0.1349 | | 8.93E-01 | |  |
| LS | Height | -0.101 | | 0.0399 | | -2.5307 | | 1.14E-02 | |  |
| CON | Height | -0.0186 | | 0.0311 | | -0.5972 | | 5.50E-01 | |  |
| AGR | Height | 0.0344 | | 0.0346 | | 0.994 | | 3.20E-01 | |  |

| Online Resource 8  Genomic SEM. | |  |  |  |  |  |  |  |  |  |
| --- | --- | --- | --- | --- | --- | --- | --- | --- | --- | --- |
| *Results EFA with all traits* | | |  |  |  |  |  |  |  |  |
| N Factors |  |  |  |  |  |  |  |  |  |  |
| **1 Factor** | **AGREE** | **CON** | **DEP** | **EXTR** | **LON** | **LS** | **NEU** | **OPEN** | **PA** | **SRH** |
| Factor 1 | -0.238 | -0.163 | 0.761 | -0.149 | 0.834 | 0.817 | 0.800 | - | 0.829 | 0.715 |
| **2 Factors** | **AGREE** | **CON** | **DEP** | **EXTR** | **LON** | **LS** | **NEU** | **OPEN** | **PA** | **SRH** |
| Factor 1 | - | - | 0.847 | 0.175 | 0.84 | 0.362 | 0.845 | - | 0.153 | 0.555 |
| Factor 2 | -0.400 | -0.190 | - | -0.396 | - | 0.567 | - | - | 0.895 | 0.221 |
| **3 Factors** | **AGREE** | **CON** | **DEP** | **EXTR** | **LON** | **LS** | **NEU** | **OPEN** | **PA** | **SRH** |
| Factor 1 | - | - | 0.815 | - | 0.792 | 0.209 | 0.982 | -0.163 | - | 0.399 |
| Factor 2 | -0.342 | -0.216 | - | -0.249 | - | 0.681 | - | 0.234 | 0.984 | -0.123 |
| Factor 3 | -0.312 | - | 0.144 | 0.652 | - | - | -0.184 | 0.578 | -0.123 | 0.178 |
| *Note.* AGREE= Agreeableness, CON= Conscientiousness, DEP=Depression, EXTR=Extraversion, LON=Loneliness, LS=Life Satisfaction, NEU=Neuroticism, OPEN=Openness to Experience, PA=Positive Affect, SRH=Self-Rated Health. | | | | | | | | | | |
|  |  |  |  |  |  |  |  |  |  |  |

| Online Resource 9  Genomic SEM. | |  |  |  |  |  |
| --- | --- | --- | --- | --- | --- | --- |
| *Factor Loadings EFA with five traits* | | | |  |  |  |
| N Factors |  |  |  |  |  |  |
| **1 Factor** | **DEP** | **LON** | **LS** | **NEU** | **PA** | **SRH** |
| Factor 1 | 0.772 | 0.843 | 0.808 | 0.806 | 0.812 | 0.721 |
| **2 Factors** | **DEP** | **LON** | **LS** | **NEU** | **PA** | **SRH** |
| Factor 1 | 0.791 | 0.694 | - | 0.996 | - | 0.358 |
| Factor 2 | - | 0.186 | 0.976 | -0.124 | 0.862 | 0.376 |
| *Note*. DEP=Depression, LON=Loneliness, LS=Life Satisfaction, NEU=Neuroticism, PA=Positive Affect, SRH= Self-Rated Health | | | | | | |
|  |  |  |  |  |  |  |
